# Transactions of the International Hahnemannian Association for 1888

**Published:** 1889-02

**Authors:** 


TRANSACTIONS OF THE INTERNATIONAL HAHNEMANNIAN ASSOCIATION FOR 1888. Edited by S. A. Kimball, M. D., Secretary, Boston.

During the past six months we have published a large number of the excellent papers read before this Association, which are now published in this volume; our readers are, therefore, well acquainted with the character of its contents. In this volume will be found articles upon nearly every subject which the practitioner of Homœopathy needs to study. Those who desire to possess a copy of these transactions can procure one of the Secretary. At page 342 an article upon *Jottings of Cases* is given, attributed to Dr. B. L. B. Baylies, which the Doctor disclaims having written!

The delay in the appearance of this volume is due to causes over which the Secretary had no proper control; another year Dr. Kimball will do himself justice and the Association greater credit.

